# Preparation of fish decalcified bone matrix and its bone repair effect in rats

**DOI:** 10.3389/fbioe.2023.1134992

**Published:** 2023-02-13

**Authors:** Zichao Liu, Xiaorui Jiang, Kai Wang, Yongshun Zhou, Tingting Li, Jianfeng Gao, Lei Wang

**Affiliations:** ^1^ College of Life Sciences, Shihezi University, Shihezi, Xinjiang, China; ^2^ Department of Hand and foot Surgery, The Affiliated Yantai Yuhuangding Hospital of Qingdao University, Yantai, China; ^3^ The Affiliated Hospital of Weifang Medical University, Yantai, China

**Keywords:** decalcified bone matrix, artificial bone material, bone defect, tissue regeneration, Hydrophilic

## Abstract

Decalcified bone matrix has great potential and application prospects in the repair of bone defects due to its good biocompatibility and osteogenic activity. In order to verify whether fish decalcified bone matrix (FDBM) has similar structure and efficacy, this study used the principle of HCl decalcification to prepare the FDBM by using fresh halibut bone as the raw material, and then degreasing, decalcifying, dehydrating and freeze-drying it. Its physicochemical properties were analyzed by scanning electron microscopy and other methods, and then its biocompatibility was tested by *in vitro* and *in vivo* experiments. At the same time, an animal model of femoral defect in rats was established, and commercially available bovine decalcified bone matrix (BDBM) was used as the control group, and the area of femoral defect in rats was filled with the two materials respectively. The changes in the implant material and the repair of the defect area were observed by various aspects such as imaging and histology, and its osteoinductive repair capacity and degradation properties were studied. The experiments showed that the FDBM is a form of biomaterial with high bone repair capacity and lower economic cost than other related materials such as bovine decalcified bone matrix. FDBM is simpler to extract and the raw materials are more abundant, which can greatly improve the utilization of marine resources. Our results show that FDBM not only has a good repair effect on bone defects, but also has good physicochemical properties, biosafety and cell adhesion, and is a promising medical biomaterial for the treatment of bone defects, which can basically meet the clinical requirements for bone tissue repair engineering materials.

## 1 Introduction

Bone tissue repair has always been a hot topic in biomedical research. In recent years, the number of patients with bone defects due to an ageing population, trauma, infections, bone tumors and congenital malformations has remained high and the demand for bone repair materials is increasing day by day (Henkel et al., 2013). Bone defects can disrupt bone continuity and lead to loss of bone function, making wound healing difficult, affecting the patient’s life and career and causing many problems for themselves and their families (Dimitriou et al., 2011). How to repair bone defects and restore normal function in a short period of time is the greatest demand of patients and the direction that the majority of medical workers are striving for.

Bone grafting or implantation of bone materials such as autologous bone, allogeneic bone and artificial bone materials are often required in the treatment of patients with bone defects. Autologous bone grafting is often seen as the best means of repairing bone defects in medicine (Pereira et al., 2017), but bone grafting operation can cause secondary injury to the body, damage the normal bone structure of the donor area and making it vulnerable to risks such as bleeding, infection and pain (De Ponte et al., 2017; Pan et al., 2018). Allogeneic bone is prone to fracture due to resorption after transplantation. On the other hand, it is also immunogenic and immune rejection and has a potential risk of epidemic transmission (Ippolito et al., 2019). In order to overcome these limitations in treatment, researchers have turned their attention to artificial bone materials with excellent properties, such as inorganic bone materials, polymer bone materials, composite bone materials and tissue engineering materials (Bian et al., 2019). Among them, biomaterials have been widely used in clinical practice for their excellent performance.

DBM is an artificial bone material obtained by decalcifying biological bone. It is a bone tissue engineering scaffold material with collagen as the main component and also contains non-collagenous proteins and lower concentrations of growth factors, with good biocompatibility and biodegradability (Hu et al., 2018; Cho et al., 2020; Hao et al., 2022). Among these, bone morphogenetic protein is the key factor in the induction of osteogenic activity, which can induce the differentiation of mesenchymal cells into chondrogenic cells and thus the formation of new bone (Chen et al., 2007; Salonius et al., 2020). The decalcification treatment removes the constraint imposed by calcium salts on bone morphogenetic protein, allowing it to be released and to fulfil its osteogenic potential (Zhang et al., 1997). Collagen is conductive to the synthesis of osteocalcin and can increase the activity level of osteoblast alkaline phosphatase. It is also conductive to the attachment of cells and the deposition of hard tissue. Loose porous structure can also promote the inward growth of osteoprogenitor cells and capillaries, so as to better play the role of bone conduction (Caballe-Serrano et al., 2020).

Currently, the DBM used in clinical practice is mainly derived from the skull, femur and tibia of terrestrial animals such as pigs, cattle, dogs and rabbits. Although they are easy to obtain, they carry a risk of transmitting diseases such as avian influenza, swine influenza and odontogenic diseases. Especially, the DBM of bovine origin carries a high risk of transmissible spongiform encephalopathy (TSE), bovine spongiform encephalopathy (BSE) and other potential viruses that may be transmitted to humans (Jonglareonrak et al., 2005; Widdowson et al., 2018). In addition to this, the DBM which derived from pigs is also banned in some countries for religious or ethical reasons. Compared with terrestrial animals, marine animals are rich in sources, easy to extract, and have higher safety. There are no animal disease risks and religious issues mentioned above (Lim et al., 2019). Marine animal collagen is similar in amino acid structure to terrestrial mammals and has a sequence structure similar to RGD amino acid sequence, which can effectively promote cell adhesion to biological materials and guide tissue regeneration, making it an ideal raw material for medical use (Silva et al., 2014; Chen et al., 2019). In addition, the presence of a large number of diaminodicarboxyl groups in the peptide chain makes the collagen of marine animals extremely hydrophilic, which can effectively solve the problem of difficult cell adhesion and proliferation on the DBM. (Wang et al., 2022).

This study was conducted to determine whether a decalcified bone matrix prepared from fish bones meets the requirements for a bone repair material and whether it has the ability to promote bone repair. In this study, fish bone from flounder was used as raw material, and the FDBM was obtained after a series of treatments, and its physicochemical properties and biocompatibility were tested. At the same time, an animal model of femoral defect in rats was established, and commercially available BDBM was used as the control group, and the area of femoral defect in rats was filled with the two materials respectively. The changes in the implant material and the repair of the defect area were observed by various aspects such as imaging and histology, and its osteoinductive repair capacity and degradation properties were studied.

## 2 Materials and methods

### 2.1 Materials

FDBM was prepared by the laboratory itself using halibut bone. BDBM was purchased from a conventional medical equipment manufacturer. Female Sprague Dawley (SD) rats (200–220 g) were purchased from Jinan Pengyue Experimental Animal Breeding Co Ltd (Jinan, China). New Zealand White rabbits (2–2.5 kg) were purchased from Qingdao Kangda Biotechnology Co Ltd (Qingdao, China). All other chemicals were of analytical grade and no further purification was required for use. All animal experiments were approved by the Animal Theory Committee of Yantai Lundy Biotechnology Co Ltd (approval number: LDSW2022037).

### 2.2 Preparation of fish decalcified bone matrix

**FIGURE 1:**
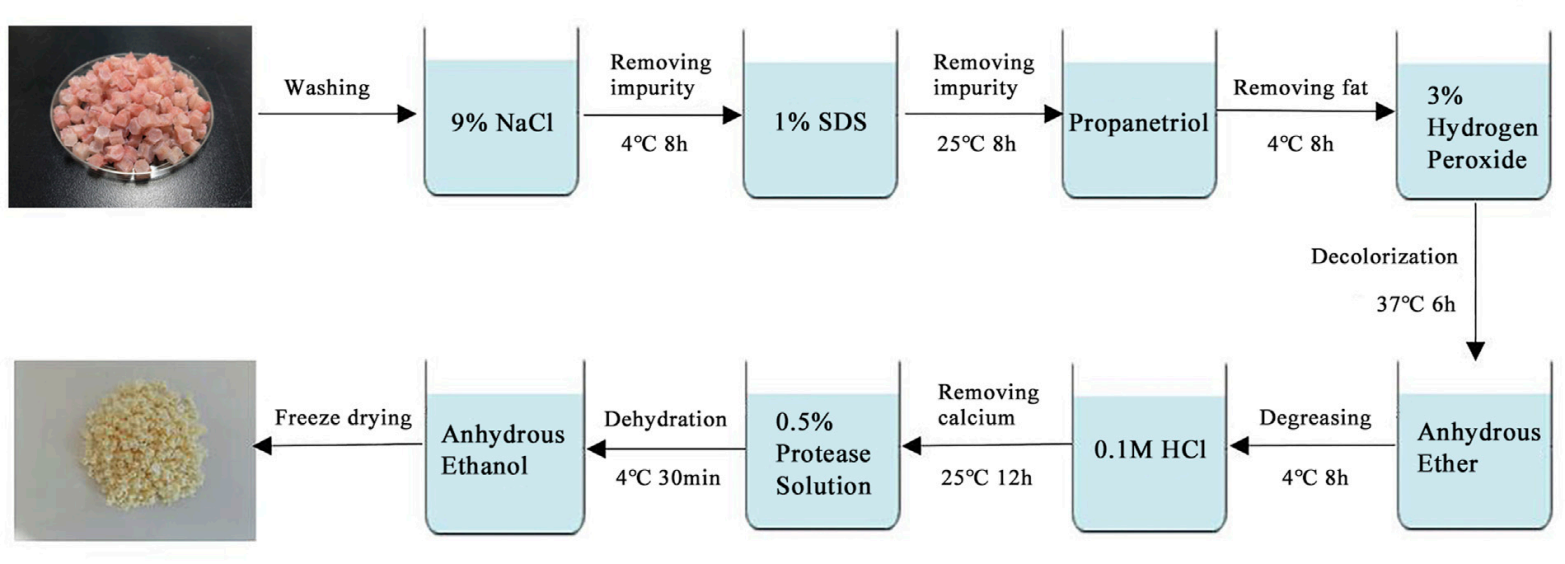
Preparation of fish decalcified bone matrix (FDBM).

This preparation process uses fresh halibut fish bones as raw material (Figure 1). We take 50 g of fish bones, cut it into 0.5 cm*0.5 cm cylinders, and rinse with pure water for 3 times. Soak the treated fish bones in 9% NaCl solution for 8 h with a material-to-liquid ratio of 1:20 (w/v) (remove impurities such as oil). The fish bones were soaked in 1% SDS solution for 8 h and the ratio of material-to-liquid was 1:20 (w/v) (remove impurities such as oil). After washing them with pure water for 3 times, the fish bones were soaked in propanetriol, and the ratio of material-to-liquid was 1:10 (w/v) (remove fat). It was stirred at 120 rpm for 8 h and filter out the solution. Then the fish bones were soaked in 3% hydrogen peroxide (H_2_O_2_) solution for 6 h, the ratio of material-to-liquid was 1:10 (w/v), and the fish bones was bleached. Wash with pure water for 3 times. The fish bones were soaked in anhydrous ether for 8 h, the ratio of material-to-liquid was 1:10 (w/v). Afterwards, the fish bones were soaked in 0.1 mol/L hydrochloric acid (HCl) and stirred with the same speed for 12 h, the ratio of material-to-liquid was 1:50 (w/v) (remove calcium). Then the fish bones were soaked in 0.5% pepsin for 30 min, the ratio of material-to-liquid was 1:30 (w/v). Finally, clean the FDBM with pure water, freeze-dry it in a freeze dryer, and sterilize it with 60Co.

### 2.3 Physical and chemical properties

#### 2.3.1 Characterisation and structural observations

The shape and morphology of the prepared FDBM was observed. Then, the structures of FDBM were observed by scanning electron microscope (SEM). The FDBM was cut into small pieces of 0.1 cm*0.1 cm and fixed on the sample table by conductive adhesive. After the surface was sprayed with gold, the surface, porosity, pore size and internal structure of the FDBM was observed by SEM (S-4800, HITACHI, Japan).

#### 2.3.2 Mechanical strength

The FDBM (cylinders of 0.5 cm diameter and 0.5 cm height) were fixed on a universal testing machine at room temperature and the specimens were crushed at a rate of 2 mm/min until the FDBM pellets were crushed into flakes (CMT8502, MTS systems, China). The compressive strength was calculated from the measured pressure values. The mean value was taken from three parallel measurements under the same conditions. The standard deviation was calculated and presented as mean ± standard deviation ( $\bar{x}\pm s$ ).

#### 2.3.3 Porosity testing

Anhydrous ethanol was added to the measuring cylinder and the volume was recorded as V_1_. The cylinder was then placed in a vacuum desiccator and evacuated to allow the ethanol to enter the pores of the material until no air bubbles escaped, at which point the volume was recorded as V_2_. Finally, the FDBM was removed and the remaining volume of ethanol was recorded as V_3_. The porosity of the FDBM was calculated according to the equation and the data are presented as mean ± standard deviation (*x̅*±s).

$$Porosity\left(\%\right)=\frac{V_{1}-V_{3}}{V_{2}-V_{3}}\times 100\%$$

In the formula: V_1_ is the initial ethanol volume; V_2_ is the total volume of FDBM after submersion in ethanol; V_3_ is the remaining ethanol volume.

#### 2.3.4 Calcium content

1) Establishment of calcium standard curve: Weigh 10 g analytically pure CaCO_3_ powder at 110°C in a beaker and gradually add 1 M HCl dropwise to the beaker until it is completely dissolved. The volume is then fixed to 100 ml in a volumetric flask. This calcium standard solution has a Ca^2+^ concentration of 1 mol/L. Dilute them into 5 parts of calcium standard solution of 1 mol/L, 0.8 mol/L, 0.6 mol/L, 0.4 mol/L and 0.2 mol/L respectively. The absorbance of the Ca^2+^ concentration of each group was observed under visible spectrophotometer 422.7 nm and the value was recorded as A. The relationship between n and A was analyzed and fitted to give the calcium standard curve equation:

$$\rho =0.9078n+0.0585\;\left(R^{2}=0.9995\right)$$

In the formula: *ρ* is the absorbance value corresponding to the concentration of Ca^2+^; n is the amount-of-substance concentration of calcium ions, mol/L.

(2) Determination of total calcium in fish bones:1 g undecalcified fish bone was placed in a beaker, 5 ml concentrated H_2_SO_4_ was added and the beaker was placed on the electrothermal furnace. Continue to raise the temperature when the fish bones are black and sticky. Gradually add HClO_4_ solution dropwise to the beaker and continue heating to make it clear and transparent. After cooling, the final volume was adjusted to 50 ml by adding deionized water. A suitable volume of liquid was taken and the absorbance was measured under visible spectrophotometer 422.7 nm. The value was recorded as A. The total calcium content of the fish bones was calculated from the regression equation in 1).
(3) Determination of calcium content in fish bone decalcification solution: A suitable volume of decalcifying solution was taken and the absorbance was measured under visible spectrophotometer 422.7 nm. The value was recorded as A. The calcium content of the fish bone decalcification solution was calculated according to the regression equation in 1).
(4) Calculation of decalcification rate:

$$Decalcification\;rate\left(\%\right)=\frac{S}{T}\times 100\%$$

In the formula: S is the calcium content in decalcification solution; T is the total calcium in fish bones.

#### 2.3.5 Water absorption

Six pieces of prepared FDBM were divided into six groups at room temperature, immersed in deionized water for 10 min and then suspended on a table. When no water drops fell from the samples, each group was weighed and the mass recorded as m. The samples were then freeze-dried, weighed again and the mass recorded as m1. Each group was repeated 3 times and the average was taken.

$$Water\;absorption\left(\%\right)=\frac{m-m_{1}}{m}\times 100\%$$

In the formula, m is the mass of FDBM after immersion in water, and m_1_ is the mass of FDBM after freeze-drying.

#### 2.3.6 *In Vitro* degradation

Six pieces of FDBM were taken, weighed and placed in a 6-well culture plate, one piece per well. 6 ml of artificial degradation solution (0.1 mol/L PBS) was added to each well, and then placed the plate in a 37°C incubator for degradation. The FDBM was removed at weeks 2, 4, 6, 8 and 10, freeze-dried and weighed, and the rate of mass loss was calculated by averaging. After each recording was completed, the physiological degradation solution was replaced with an equal amount to continue the degradation. The degradation rate of the FDBM was calculated for each time period (2 weeks for each time period) and a degradation rate curve was plotted.

$$Degradation\;rate\left(\%\right)=\frac{A_{0}-A_{1}}{A_{0}}\times 100\%$$

In the formula, A_0_ is the mass of FDBM at one time point before degradation, and A_1_ is the mass of FDBM at one time point after degradation.

### 2.4 Biocompatibility

#### 2.4.1 Cytocompatibility

In order to evaluate the cytotoxicity, proliferation rate and adhesion of FDBM, we chose mouse fibroblasts (L929) as co-cultured cells with FDBM.

##### 2.4.1.1 Cytotoxicity

Under aseptic condition, 5 g FDBM were placed in 30 ml DMEM high glucose (DMEM-H) complete medium and extracted at 37°C with 80 rpm for 24 h to obtain cell culture medium. The cytotoxicity was detected by CCK-8 method. L929 cells are cultured with the DMEM-H system containing 10% FBS. According to the standard of 8×10^3^/well, L929 cells at logarithmic growth stage were inoculated in a 96-well plate with a volume of 100 μL per well. The upper layer of the cell culture medium was discarded after the cells adhered to the wall and formed monolayers. Then the mixture of 200 μl cell culture medium and extraction solution were sequentially added to the 96-well plate according to the proportion of 25, 50%, and 100%. After co-culture with cells for 24 h, each well was equipped with 100 μl CCK-8 solution (10 μl CCK-8 in 90 μl medium) according to the instructions of CCK-8 kit (Biosharp, China). Then the culture plate was incubated at 37°C and 5% CO2 for 2 h, and the optical density (OD) value was detected at 450 nm by microplate reader (Infinite F50, Tecan, Switzerland).

$$Cell\;proliferation\;rate\left(\%\right)=\frac{A_{s}-A_{b}}{A_{c}-A_{b}}\times 100\%$$

In the formula, A_s_ is the absorbance of wells with cells, CCK8 solution and leachate, A_c_ is the absorbance of wells with cells, CCK8 solution and no extraction solution, and A_b_ is the absorbance of wells with medium and CCK8 solution and no cells.

##### 2.4.1.2 Live/Dead Cell staining

According to the standard of 1×10^5^ cells per well, 500 μL cell suspension was put into 48-well plate. After the cells adhered to the wall, the original cell culture medium was replaced with the extraction solution. After culturing for 3 days, replace the normal cell culture medium with 1.5 μL propidium iodide and 1 μL calcein-AM (Solarbio, China) in PBS solution, and incubate for another 30 min. After the sample was gently washed with PBS, fluorescence was excited by 490 nm wavelength under inverted fluorescence microscope (ECHO RVL-100-G, United States), and the distribution of living dead cells was observed.

##### 2.4.1.3 Observation of Cell adhesion

L929 cells were cultured in a cell incubator at 37°C and 5% CO2. The cells were digested and counted when the cells grew to the logarithmic phase. After 1 ml cell suspension was inserted into a 24-well plate. FDBM was put into the cell suspension, co-cultured with the cells for 24 h, fixed with 2.5% glutaraldehyde solution for 24 h and lyophilized. The cells were observed for adhesion, infiltration and growth on the surface and inside the bone material by scanning electron microscope.

#### 2.4.2 *In Vivo* safety and degradation

##### 2.4.2.1 In vivo implantation

Twelve SD rats were randomly divided into four groups and each group consists of seven rats. After the rats were anesthetized with 10% chloral hydrate at a dose of 0.4ml/100g, their backs were depilated within the 1 cm*1 cm area. After alcohol disinfection, the full-thickness skin openings of 0.5 cm were opened at 1.5 cm on both sides of the dorsal spine. Then FDBM and BDBM were placed on the left and right sides, respectively, and sutured. After all rats were awakened, the rats were observed for their living condition, mental status and feeding.

##### 2.4.2.2 Histopathological Examination

On the 3rd, 7th, 14th, and 28th day after the operation, one group of rats were sacrificed respectively, and the embedded FDBM and BDBM were took out, which were fixed with 4% paraformaldehyde. After 24 h of fixation, the tissues were embedded in paraffin and sectioned in routine paraffin for HE and Masson staining. Finally, the morphology and cell infiltration of FDBM and BDBM were observed under light microscope.

#### 2.4.3 Haemolysis rate

Under aseptic condition, 5 g FDBM were placed in 30 ml normal saline and extracted at 37°C with 80 rpm for 24 h to obtain the extraction solution of FDBM. 2 ml fresh anticoagulant blood was taken from healthy SD rats and 8 ml normal saline was added to dilute it. 10 ml FDBM extraction solution were prepared as experimental group. At the same time, the same amount of deionized water was used as the positive control group and the same amount of normal saline as the negative control group. Then 200 μl SD rats’ blood was added to test tubes from three groups, which are mixed thoroughly and incubated at 37°C for 60 min. After incubation, the tubes were centrifuged at 2500 rpm speed for 5 min in a high-speed centrifuge. After centrifugation, the hemolysis was observed, and the absorbance was measured with an ultraviolet spectrophotometer at 545 nm wavelength.

$$Haemolysis\;rate\left(\%\right)=\frac{A_{1}-A_{2}}{A_{3}-A_{2}}\times 100\%$$

In the formula, A_1_ is the average absorbance of FDBM group, A_2_ is the average absorbance of normal saline group, and A_3_ is the average absorbance of deionized water group.

#### 2.4.4 Pyrogen testing

The FDBM was tested for pyrogen by reference to the Chinese Pharmacopoeia (2020 edition). The extraction solution of FDBM was obtained by the method in 2.4.3, placed in a water bath and preheated to 38°C. Then three normal New Zealand White rabbits were taken and their body temperature was measured. 25 ml extraction solution of FDBM was slowly injected from each rabbit’s ear marginal vein, and then the temperature change was measured in real time over a 3 h period using the same online real-time thermometer. After the measurement was complete, the highest temperature during the entire test was used to subtract the normal body temperature of the rabbit as the number of degrees of increase in body temperature for this test. If all temperatures measured throughout are lower than normal body temperature, record as 0°C.

### 2.5 Ability to repair bone defects

#### 2.5.1 Establishment of bone defects model

Forty-five SPF-grade 10-week-old female rats (200–220 g) were randomly divided into three groups of fifteen rats each. 10% chloral hydrate was injected intraperitoneally according to 0.4 ml/100 g body weight ratio. Aseptic operation was maintained during the operation. After rats were placed in a supine position, the hair on the inner side of the leg was removed and the skin was exposed and disinfected with 75% alcohol. Then a 1 cm skin incision was made along the left femur, the skin and muscle layers were spread along the femur in turn to expose the femoral stem. The periosteum was then separated to expose the middle and upper part of the femoral stem. The rat’s femur was drilled with a 2 mm diameter drill, the drill was moved up and down to create a bone defect area of approximately 2 × 3 mm^2^. The holes were flushed with sterile saline to quickly remove the bone fragments.

After the bone defects model was prepared, FDBM and BDBM were implanted into the bone defect area of different group, and the defect area was adequately filled. The self-healing group did not do any treatment. After implantation, the wound was closed with sutures and 400,000 units of penicillin were injected into the other thigh muscle to prevent infection. Normal feeding after operation.

#### 2.5.2 Postoperative status observation

After the operation, the rats’ spirit, feeding, activity and wound healing were recorded daily. The effect of the surgery and the implant material on the rats was judged according to their behavior and reaction status. At the same time, the occurrence of redness, swelling, oozing and pus at the operative site was recorded. Five rats in each group were euthanized at the fourth, 8th and 12th weeks after operation, and the whole femur was removed. The muscles, fascia and other tissues on the femur were cleared and then the bone defect area was observed for the state of the material, the state of healing and the presence of infection.

#### 2.5.3 CT radiographic observation

The femur in 2.4.2 was fixed in 4% paraformaldehyde for 24 h. The specimen was removed and washed three times with PBS buffer (pH 7.0) before CT radiography was performed to observe the bone repair of the defect site and the degradation of the implant material.

#### 2.5.4 Histopathological examination

The rat femurs were put into the EDTA decalcification solution and placed in a constant temperature shaker at 37°C, 50 rpm for 1 month, during which time the EDTA decalcification solution was changed once a week. After decalcification was completed, the rat femurs were washed with distilled water, routinely dehydrated, embedded in paraffin, sectioned and then subjected to HE and Masson staining. The ability of the bone material to repair bone defects was evaluated by observing the degradation of the bone materials, the cell type and status of the bone defect area, and the bone repair at the defect site. New bone formation was evaluated semi-quantitatively at the 4-week time point with reference to the histological outcome assessment criteria in “YY/T 1680–2020 *In vivo* evaluation of osteoinductive potential for materials containing demineralized bone”. The results were evaluated independently by two investigators blinded and counted.

## 3 Result

### 3.1 Physical and chemical properties of FDBM

The FDBM was prepared from flounder fish bones through a process of defatting, decalcifying and freeze-drying. The freeze-dried FDBM is faintly yellow, hard and dense. As a whole, its shape resembles that of a cylinder with a diameter and height of 0.5 cm. From the side, its shape is irregular, with two large ends and a thin middle part. In the determination of calcium content in FDBM, the relationship between n and A was analyzed and fitted to give the calcium standard curve equation: ρ = 0.9078n + 0.0585 (R^2^ = 0.9995). In the formula: ρ is the absorbance value corresponding to the concentration of Ca^2+^; n is the amount-of-substance concentration of calcium ions, mol/L. After calculation, the average decalcification rate of FDBM is 78.41 ± 5.73%. Normally DBM has the disadvantage of poor mechanical properties, so we did not completely decalcify it during the preparation of FDBM. This approach allows the FDBM to maintain good mechanical strength without compromising the repair effect. This facilitates the better use of FDBM in bone tissue engineering. In the process of tissue repair, materials with higher porosity can provide a wider space for cell adhesion, proliferation and differentiation. The results show that the porosity of FDBM is 72.56 ± 4.67%, which basically meets the requirements of an ideal bone tissue engineering material.

**FIGURE 2:**
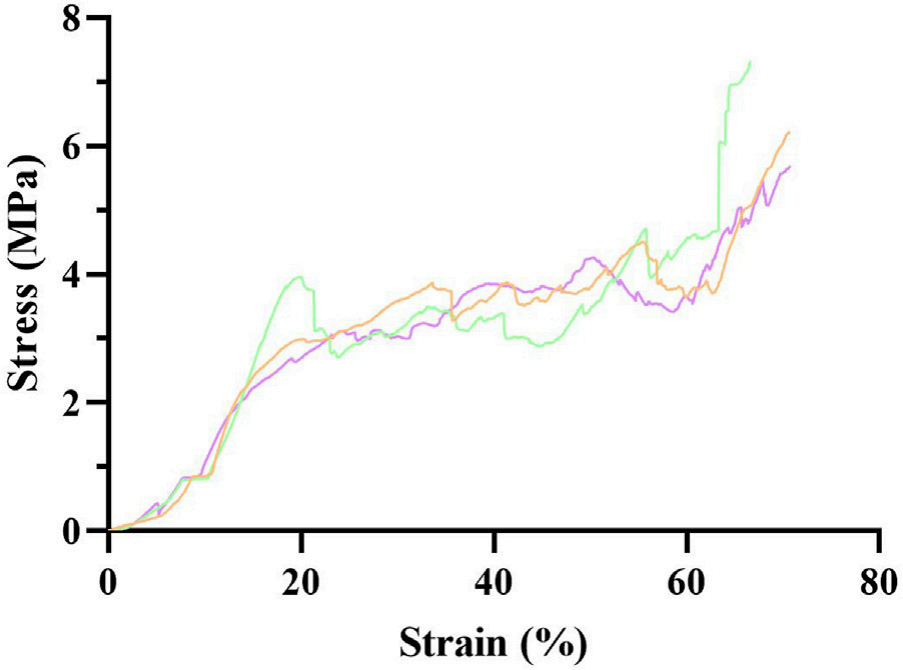
Mechanical strength diagram of FDBM (n = 3)

The mechanical strength of the FDBM was measured using the compression test method and the first turning point of the compression curve was defined as the mechanical strength. Figure 2 shows that the stress increases as the FDBM is continuously compressed, with a turning point when the strain reaches about 30%. The compressive stress when FDBM was destroyed was 4.03 ± 0.17 MPa which is close to the strength of human cancellous bone (2–20 MPa) (Bose et al., 2012). The results indicating that FDBM has excellent mechanical strength and is very suitable for biomedical materials.

**FIGURE 3:**
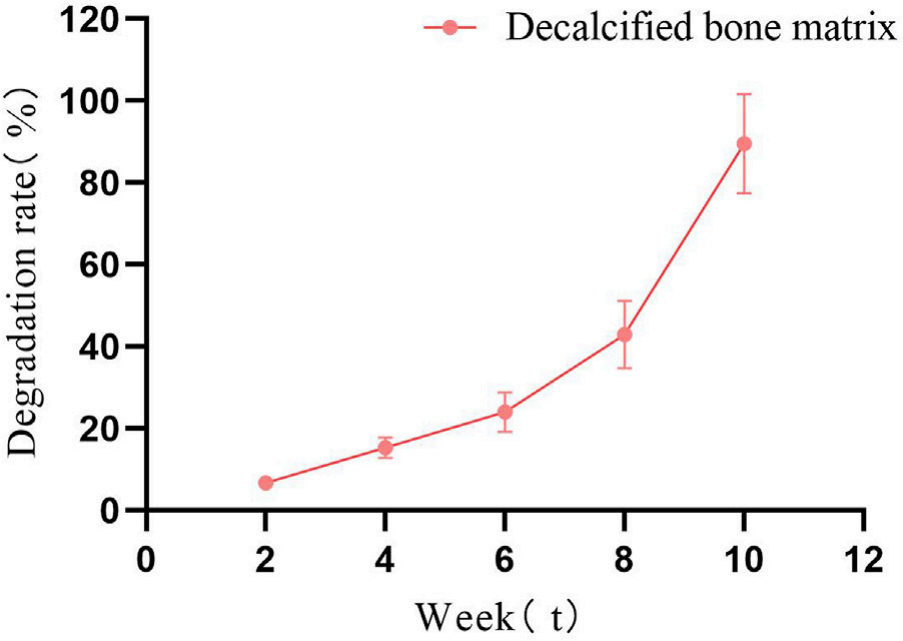
*In vitro* degradation diagram of FDBM (The test was conducted for a total of 10 weeks, with observations every fortnight.) (n = 6).

The hydrophilicity of biomaterials is an important parameter in tissue engineering applications, and good hydrophilicity facilitates cell adhesion, growth and differentiation (Mishra et al., 2019). HCl treatment can fully expose the hydrophilic groups of fish bone, so that the FDBM has good hydrophilicity. Experiments showed that the water absorption rate of the FDBM can reach 86.41% (Table 1). The ideal bone tissue engineering scaffold should also have good biodegradability. The degradation rate of the scaffold after implantation should be commensurate with the growth rate of the tissue and should maintain its shape over a period of time, which can provide a shaping effect on the new tissue. Too fast or too slow a degradation rate of the scaffold can affect the structure of the new tissue. Figure 3 shows that the FDBM degrades *in vitro* in a physiological degradation solution at a slow rate for the first 6 weeks, with a significantly faster rate after 6 weeks and rapid cleavage as it approaches 10 weeks. The results show that the *in vitro* degradation time of FDBM is approximately 8–10 weeks.

**TABLE 1:** Water absorption in each group (n = 3, per group).

Groups	Before absorbing water ( $\bar{x}$ ±SD)	After absorbing water ( $\bar{x}$ ±SD)	Water absorption (%)
1	0.0351 ± 0.0026	0.0674 ± 0.0046	92.11
2	0.0324 ± 0.0027	0.0604 ± 0.0045	86.47
3	0.0273 ± 0.0033	0.0515 ± 0.0051	88.83
4	0.0311 ± 0.0025	0.0572 ± 0.0047	83.72
5	0.0333 ± 0.0024	0.0590 ± 0.0049	77.31
6	0.0316 ± 0.0032	0.0602 ± 0.0065	90.30

**FIGURE 4:**
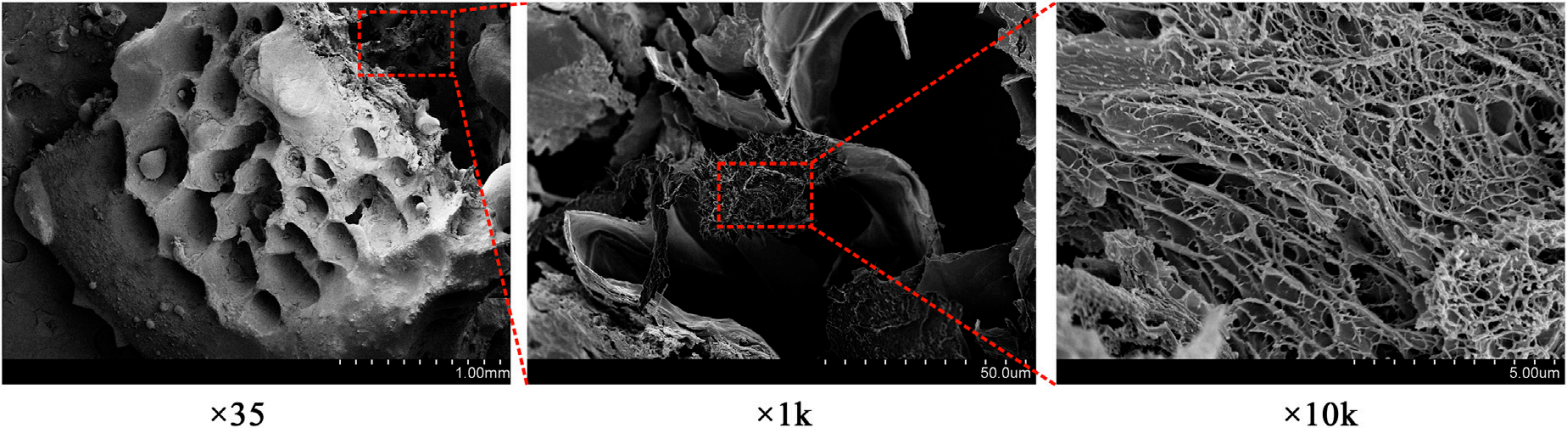
Electron microscope scan of FDBM.

The ideal bone repair scaffold should have a highly interconnected porous structure that provides a biological environment conductive to cell adhesion and proliferation as well as tissue growth and nutrient flow (Zhang et al., 2019). Figure 4 shows that the freeze-dried FDBM showed a porous honeycomb shape with a dense and regular arrangement in the electron microscopic field of view. At high magnification, the FDBM shows a loose and porous structure with good connectivity between the porous structures. The pores of the FDBM range from 10 μm to 50 μm, and its loose and porous structure provides good access and storage for small molecules, which provides a structural basis for cell crawling and growth inside the scaffold (Lv et al., 2021).

### 3.2 Biocompatibility assessment of FDBM

#### 3.2.1 Cytocompatibility

**FIGURE 5:**
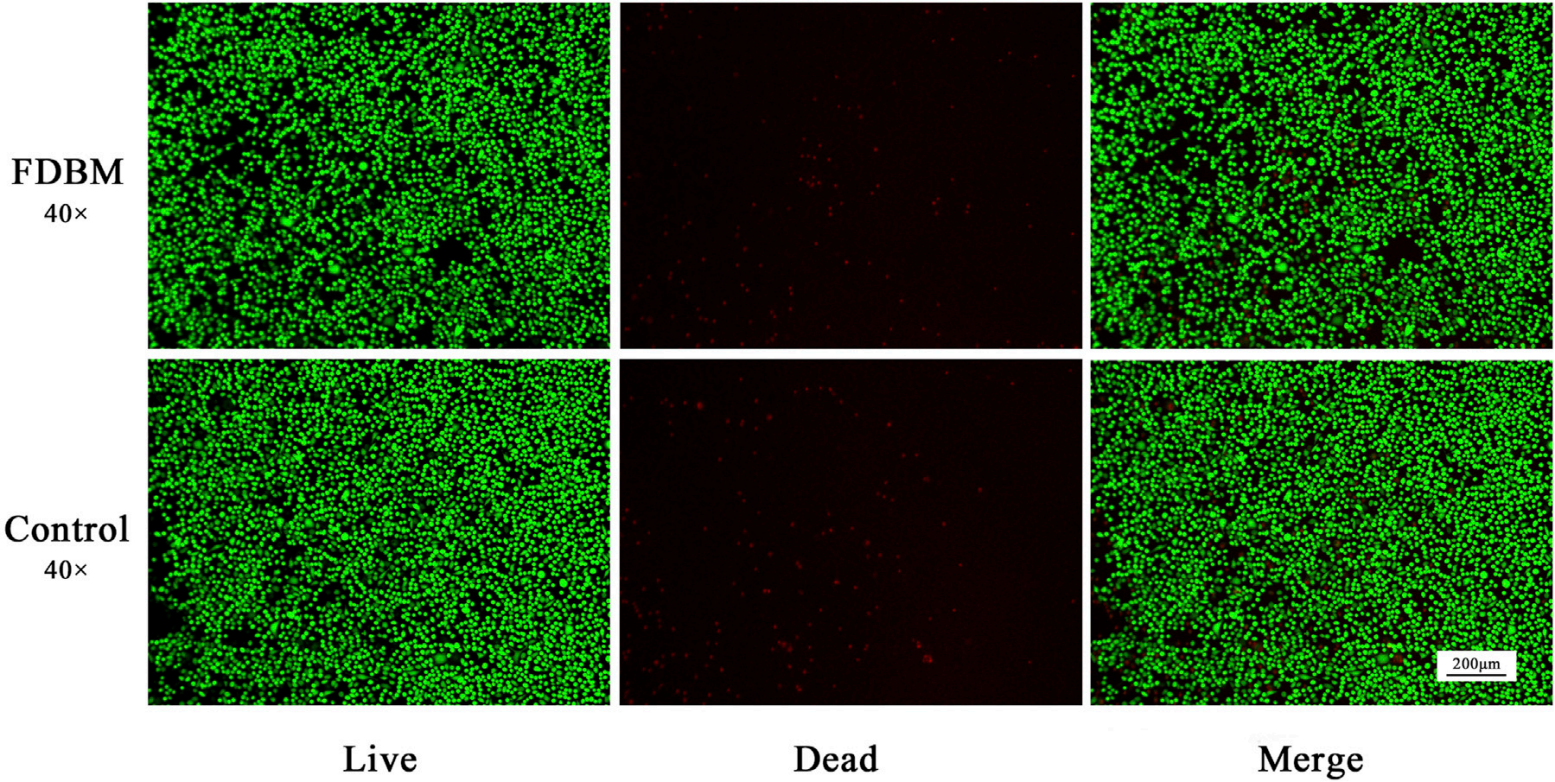
Live-Dead staining of L929 cells cultured by FDBM extraction solution after culturing for 3 days (green for living cells and red for dead cells; magnification, ×40).

**FIGURE 6:**
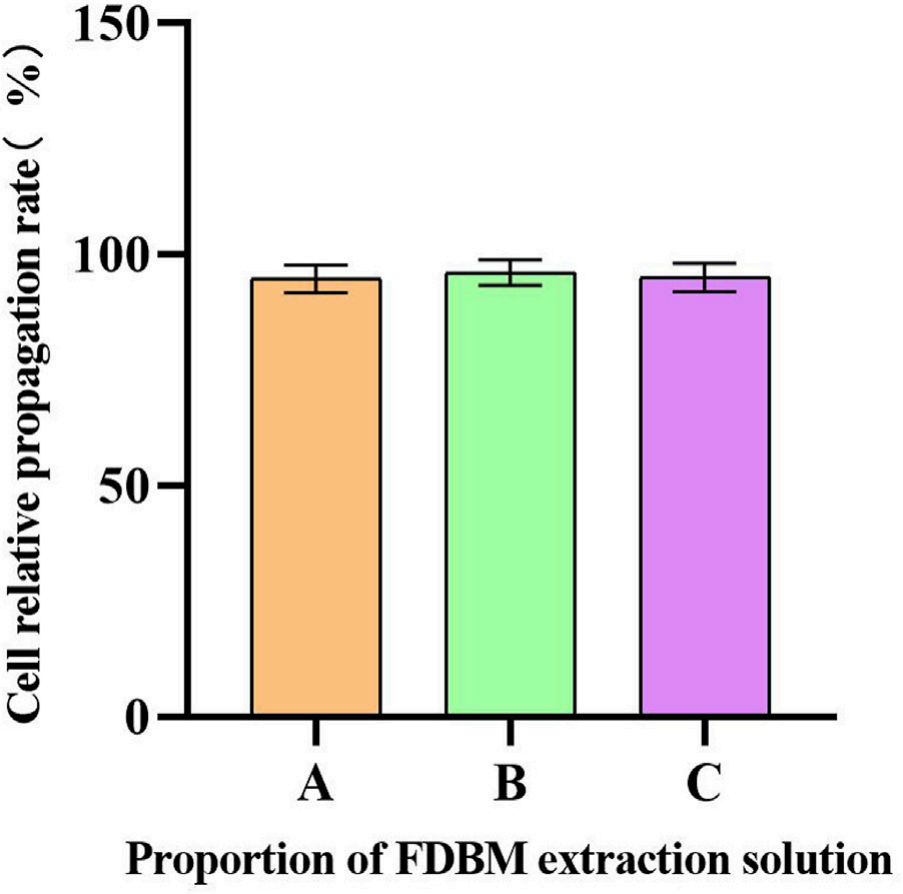
Cell proliferation rate. The horizontal coordinate is the proportion of FDBM extraction solution to the cell culture medium. A is 100%; B is 50%; C is 25%. (There was no significant difference between the three data sets.) (n = 6).

Cell metabolism can be affected by cytotoxic materials. After the Calcein AM-PI staining, the living cells were green and the dead cells were red (Zhang et al., 2021). From the staining results of live/dead cells cultured to 3 days (Figure 5), the cells cultured by FDBM extraction solution grow well compared with the control group, and there is no significant difference in the proportion of dead cells, indicating that the FDBM scaffold has no cytotoxicity. Similarly, the results of CCK-8 test (Figure 6) also proved that the prepared FDBM is non-cytotoxic. The cells cultured in all three concentrations of FDBM extraction solution had a cell proliferation rate of around 95%. The cellular value-added rates at the three concentration tests were approximately equal and the differences were not statistically significant. According to the grading standard of cytotoxicity evaluation, the cytotoxicity is level 1. According to the standard, it is deemed to be non-cytotoxic when the cytotoxicity is at the first level. The cytocompatibility of TADM was satisfactory.

**FIGURE 7:**
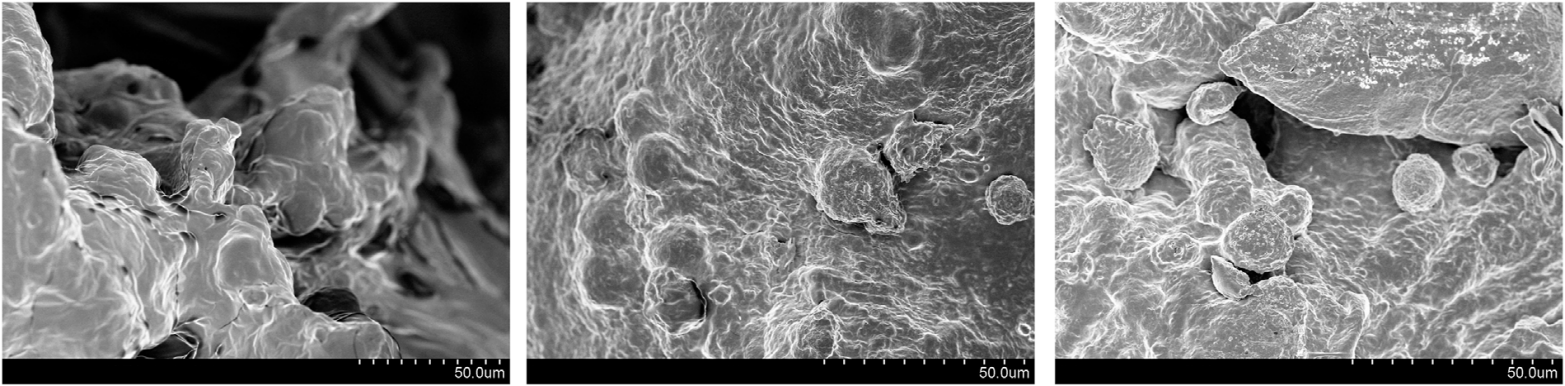
Electron microscope picture of the adhesion and growth of L929 cells on FDBM.

Figure 7 shows that L929 cells can normally infiltrate the FDBM and adhere to it. A large number of cells adhere to the interior of the material, but the cells mainly adhere to the collagen surface and extend pseudopods on its surface, showing some crawling behavior. In addition, some of the cells replicate and proliferate inside the material. This again demonstrates the good cytocompatibility of the FDBM. The results show that the FDBM not only has high porosity, but also has good fibroblast adhesion and potential to induce fibroblast migration and growth, which fully indicates that the FDBM has good cell compatibility.

#### 3.2.2 Destructive effect on blood cells

**FIGURE 8:**
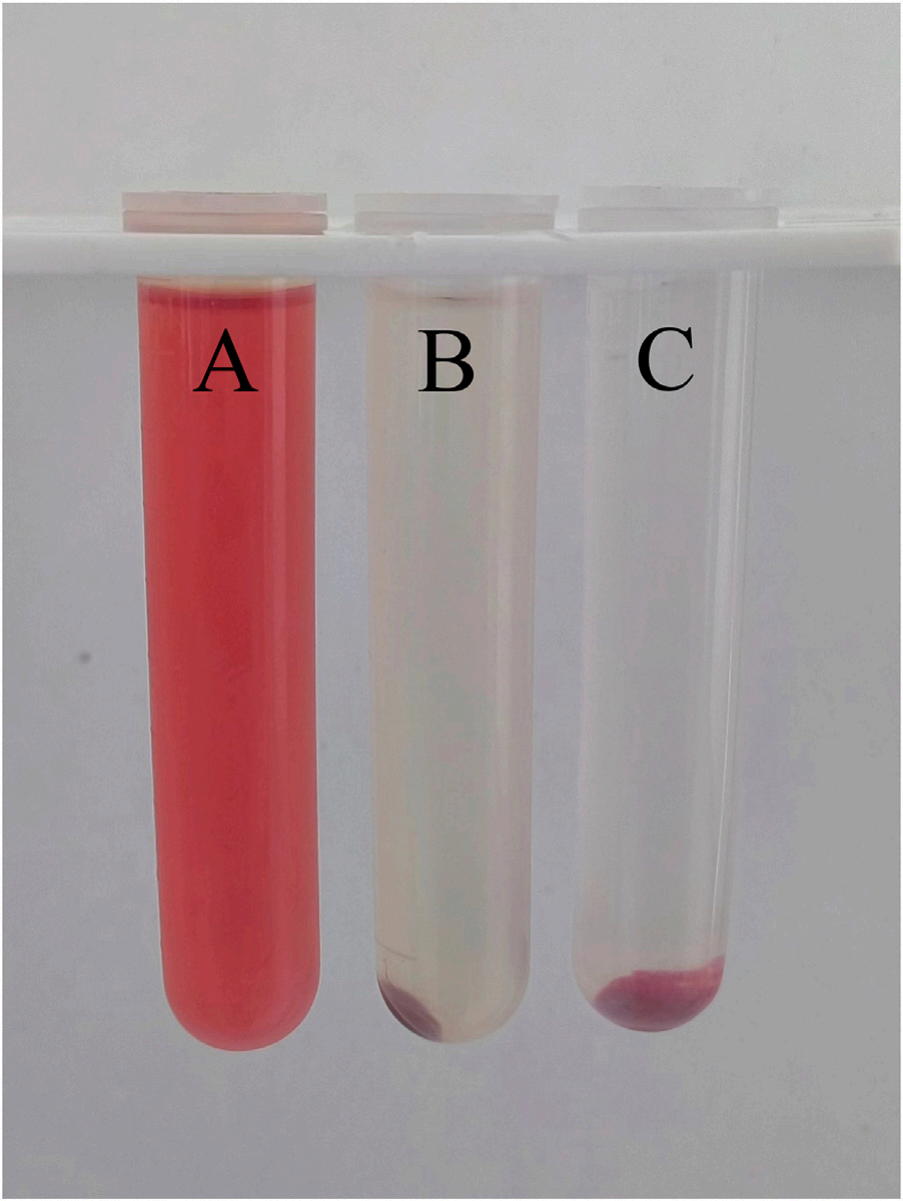
The result diagram of FDBM hemolysis (A is distilled water, B is FDBM extraction solution, C is normal saline) (n = 10 per group).

The results of the hemolysis test (Figure 8) showed that the supernatant of the normal saline group was clear and transparent, and no hemolysis occurred. The supernatant of the FDBM group was slightly red, and most of the red blood cells were precipitated to the bottom of the tube, only a very small number of red blood cells were lysed. The distilled water group had no red blood cell precipitation, and the liquid in the tube appeared uniformly red. The absorbance was measured by adjusting the wavelength of the UV spectrophotometer to 545 nm. General standards stipulate that if hemolysis rate is less than 5%, the material could meet the clinical blood safety requirements. The average hemolysis rate of the FDBM group was only 1.55% (Table 2), which was much less than 5%. It can be determined that it will not cause a hemolytic reaction.

**TABLE 2:** OD and hemolysis rate in each group (n = 10, per group).

Groups	OD ( $\bar{x}$ ±SD)	Average hemolysis rate (%)
Fish decalcified bone matrix (FDBM)	0.031 ± 0.004	1.55
Physiological saline (NS)	0.012 ± 0.002	-
Distilled water (DI)	1.235 ± 0.005	-

#### 3.2.3 Pyrogen testing

**FIGURE 9:**
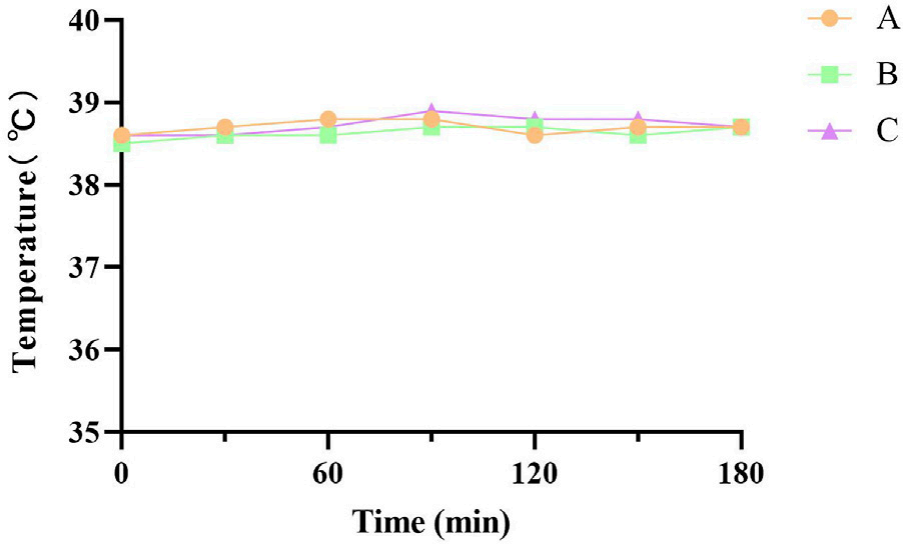
Temperature test curve of New Zealand white rabbits. A, B and C are tests on three New Zealand White rabbits (Test every 30 min Six tests in total.).

The entry of substances with immunogenicity into the body induces an immune rejection reaction, which is manifested externally by increased body temperature and poor mental status (Gil-Castell et al., 2020). All New Zealand White rabbits were in good spirits and could eat normally without any abnormal reaction after the injection of the FDBM extraction solution. Body temperature testing (Figure 9) showed that the rabbits’ temperature had no significant change at a total of 6 time points over a period of 3 h after injection. All rabbits did not have an increase in body temperature of more than 0.6°C and the sum did not exceed 1.3°C. The results showed that the FDBM was free of pyrogenic reactions and complied with the provisions of the standard results composite related to pyrogenic reactions of biomedical materials.

#### 3.2.4 Degradation in the body

**FIGURE 10:**
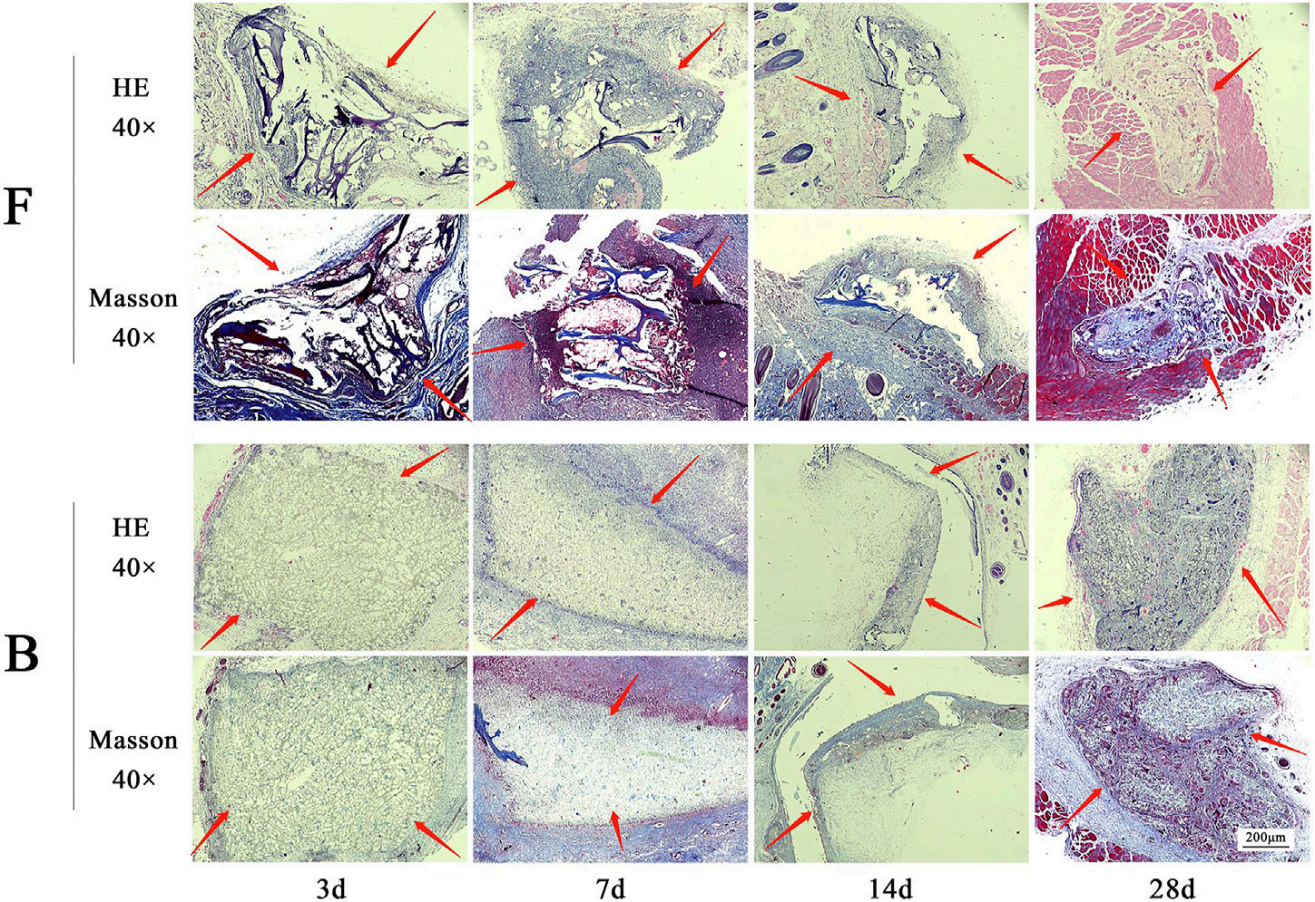
The results of HE and Masson staining are obtained by subcutaneously embedded FDBM and BDBM. The tissues were taken for H&E and Masson staining on the third, seventh, 14th and 28th day after implantation (magnification, ×40). F is the FDBM group, B is the BDBM group (The red arrows show the implanted bone matrix).

After the DBM was buried subcutaneously in the rats, the rats lived in good condition and ate normally during the observation period. There was no immune rejection and allergic reactions such as swelling, redness, or seeping pus in the area of the implantation. The histological staining of subcutaneously implanted DBM is shown in Figure 10. The results showed that both groups of material were encapsulated by the fibrous capsule wall on day 3, along with a large infiltration of tissue fluid. There were some inflammatory cells entering the scaffold along the larger pores. On day 7, a small number of inflammatory cells have infiltrated the pores of the scaffold, and the collagen fibers in the scaffold have begun to degrade and adhere to the connective tissue of the skin. On day 14, the fibrous capsule that encases the material was significantly smaller. The collagen fibrous structure became incomplete and gradually fused with the skin and cells as with normal soft tissue. On day 28, the volume of the remaining bone material was approximately 1/2 of the initial volume and degradation was evident. The inflammatory cells largely disappeared. The results show that the fish decalcified bone matrix has good biocompatibility and does not cause inflammatory reactions or immune rejection in rats. Its good *in vivo* degradability is also demonstrated.

### 3.3 Femoral defect repair in rats

#### 3.3.1 Postoperative status observation

**FIGURE 11:**
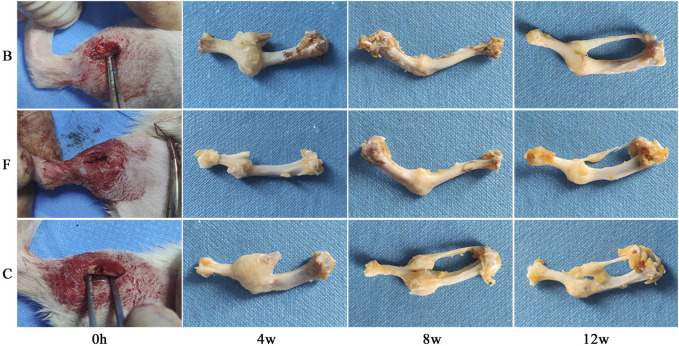
Effect of repairing femoral defect (B: BDBM group; F: FDBM group; C: Self-healing group).

All the rats in the bone defect model woke up within 1 h after surgery, and their mental and dietary conditions were slightly poor for the first 3 days, and returned to normal after 3 days. During the whole experimental cycle, the surgical area of the rats healed well and no obvious symptoms of infection such as swelling, redness, or seeping pus were observed. Figure 11 showed that all three groups of rats had formed a larger volume of bone crust structure at the site of the bone defect at week 4 postoperatively. Among them, the self-healing group had the largest bone crust structure, and the FDBM group had a smaller bone crust structure compared with the BDBM group. The area of the femoral defect was significantly reduced in all three groups, with the FDBM group showing the greatest reduction and the self-healing group the smallest. At 8 weeks postoperatively, the bone defect area in the FDBM group was almost completely healed and the bone surface had been repaired, but the bone crust structure was still present and significantly smaller than at 4 weeks. The repair status of the BDBM group was similar to that of the FDBM group, but the bone crust structure was relatively larger. The bone defect area in the self-healing group was not completely healed in all rats, and the remaining defect area was significantly larger than the other two groups, and the bone crust structure was still significantly. At 12 weeks postoperatively, the FDBM group had the best healing status, with no capsule or encapsulation around the material. The defect surface was largely intact, with the bone crust structure largely gone. The bone defect area in the BDBM group also healed better, but there was still a relatively small bone crust structure in all rats. The defect area in the self-healing group was not repaired, and the bone crust structure was significantly larger in all rats than in the other two groups.

From this, it can be tentatively judged that the prepared FDBM has good ability to induce bone repair and that it repairs bone defects faster than commercially available BDBM. It can be used as a potential clinical bone repair material.

#### 3.3.2 CT radiographic observation

**FIGURE 12:**
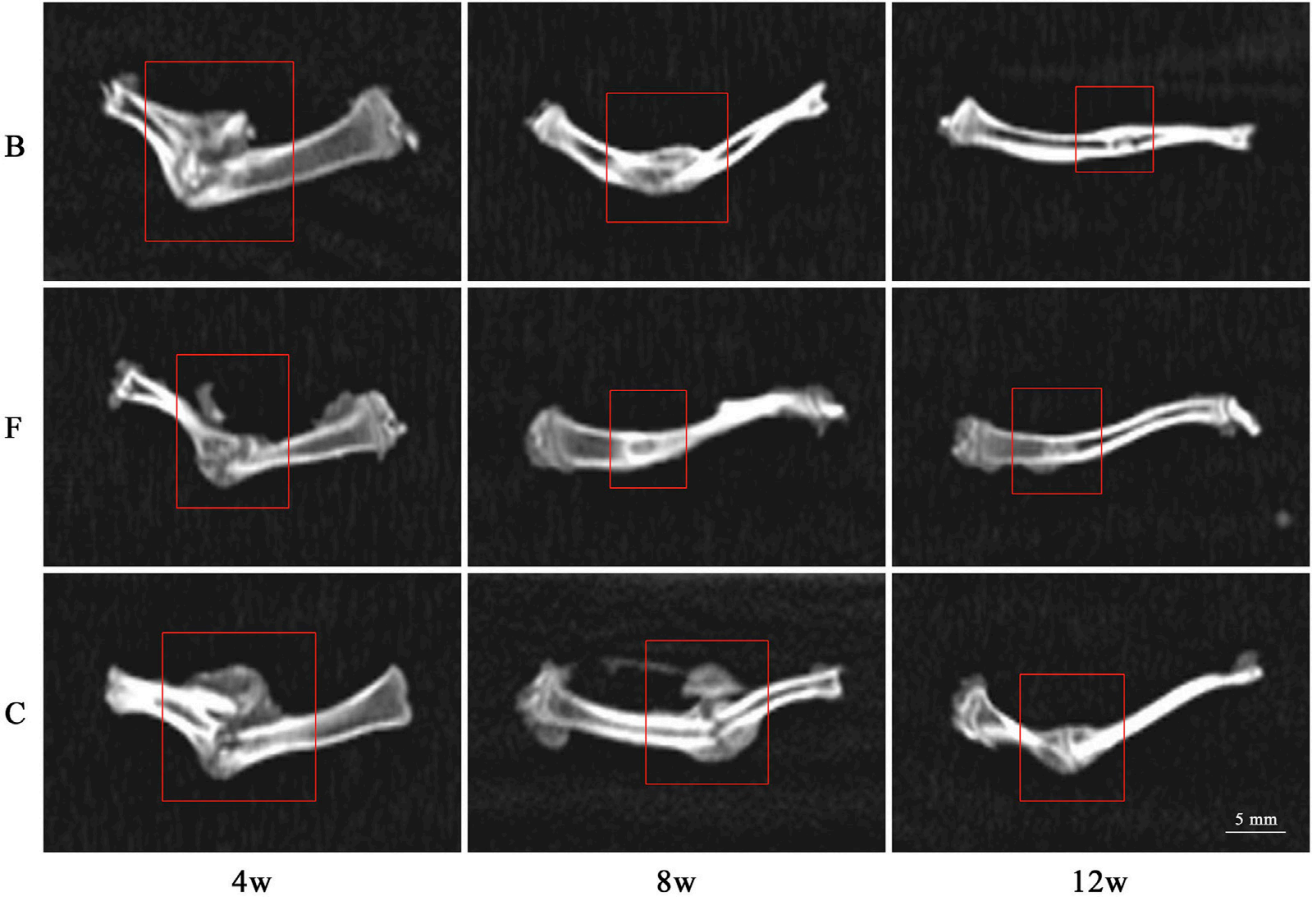
CT radiographic observation (B: BDBM group; F: FDBM group; C: Self-healing group).

The femurs of the rats removed in 3.3.1 were subjected to CT radiographs and the image data were analyzed to observe bone repair at the defect site and degradation of the implant material (Figure 12). At week 4 postoperatively, both the self-healing group and the group filled with bone material showed varying degrees of fracture at the site of the bone defect. The edges of the defect area were clear and the size of the defect was not significantly reduced. The bone material in both groups showed a hypodense shadow that could be completely visualized and differed significantly from normal femoral tissue. There was no new bone production on the marginal bone surface of the material and the femoral defect area.

At week 8 postoperatively, CT images showed varying degrees of shrinkage of the bone crust in the area of the bone defect in all three groups. In the self-healing group, the bone scab structure was the largest, and the FDBM group had a smaller bone scab structure compared to the BDBM group. In addition, the area of hypodense shadow was significantly reduced in the FDBM group, and the density of the defect area was close to that of normal femoral tissue, indicating that new bone tissue was being produced along the edges of the defect area towards the center, but complete healing of the entire defect area had not yet been achieved. In contrast, the femoral defect area in the BDBM group recovered slightly less well.

At week 12 postoperatively, only about 1/4 of the defect area in the FDBM group was slightly less dense than the high-density area, while the rest of the area was not significantly different from normal femoral tissue. This indicates that the FDBM has been resorbed, degraded and mature new bone tissue has formed at the edges and within the defect area. There is no longer any bone crust structure present on the image. The difference between the BDBM group and the FDBM group was not significant, with only a small portion of the defective area being slightly less dense than the high-density area, while the rest of the repaired defective area did not differ significantly in bone structure and density from the normal area. The self-healing group still had a large amount of bone scab structure present and the defect area showed a large hypodense shadow, mostly not replaced by new bone tissue, with poor recovery.

The CT results showed that the FDBM was superior to the BDBM in terms of speed of bone healing and denseness of the bone tissue formed, indicating that the FDBM has good osteogenic ability and is a good material for bone repair.

#### 3.3.3 Histopathological analysis

**FIGURE 13:**
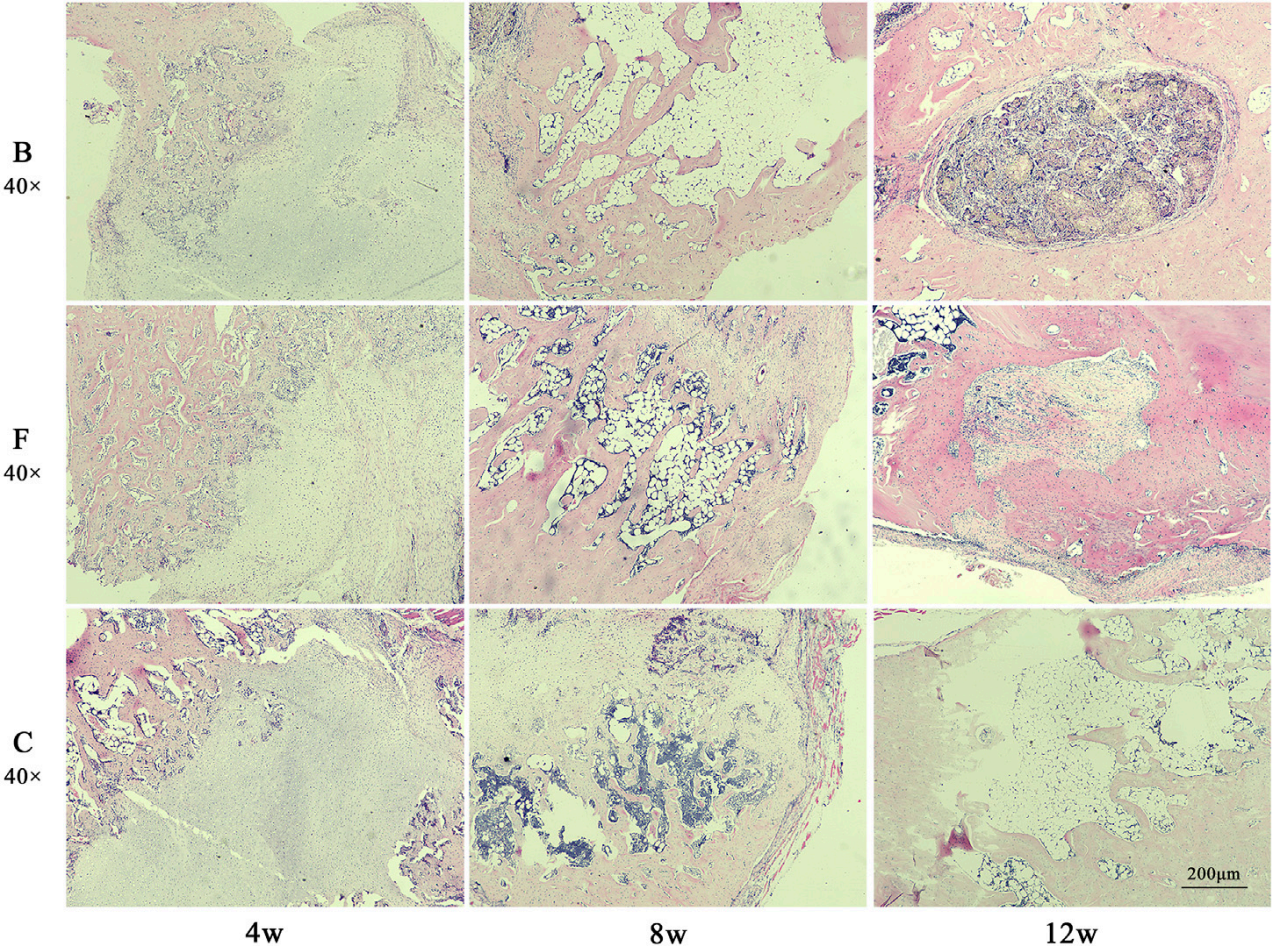
The H&E staining map of bone defects in rats. The tissues were taken for H&E staining at weeks 4, 8 and 12 after implantation (magnification, ×40).

The results of HE staining (Figure 13) showed that the FDBM group had been partially degraded at week 4 and the remaining material was wrapped in fibrous tissue. Newly generated bone tissue and bone trabeculae were interspersed between and at the edges of the fibrous tissue. There were also a number of inflammatory cells and osteoblasts distributed in the fibrous tissue. At week 8 postoperatively, the interior of the bone defect was covered with new bone tissue. The osteocytes are distributed among the newly created bone tissue and there are no inflammatory cells. There was still a small amount of artificial bone material located in the middle of the new bone tissue. At week 12 postoperatively, the entire defect area had been filled with new bone tissue and there was a significant number of osteoblasts present in the bone tissue. The area was fully integrated with the original femoral tissue, except for a small intermediate area which was slightly less integrated with the surrounding bone tissue. The BDBM group showed the same trend of bone repair as the FDBM group at the first two time points. However, at week 12, a small portion of the defective area was still filled with collagen fibers and was not fully osteogenic. The repair effect was slightly lower than in the FDBM group. The self-healing group had the worst bone repair effect, with significantly worse bone trabeculae numbers and new bone tissue density at each time point than the FDBM group and the BDBM group. At week 12, the interior of the defect area was still relatively sparse with new bone tissue and fibrous tissue, and large areas were not completely filled.

**FIGURE 14:**
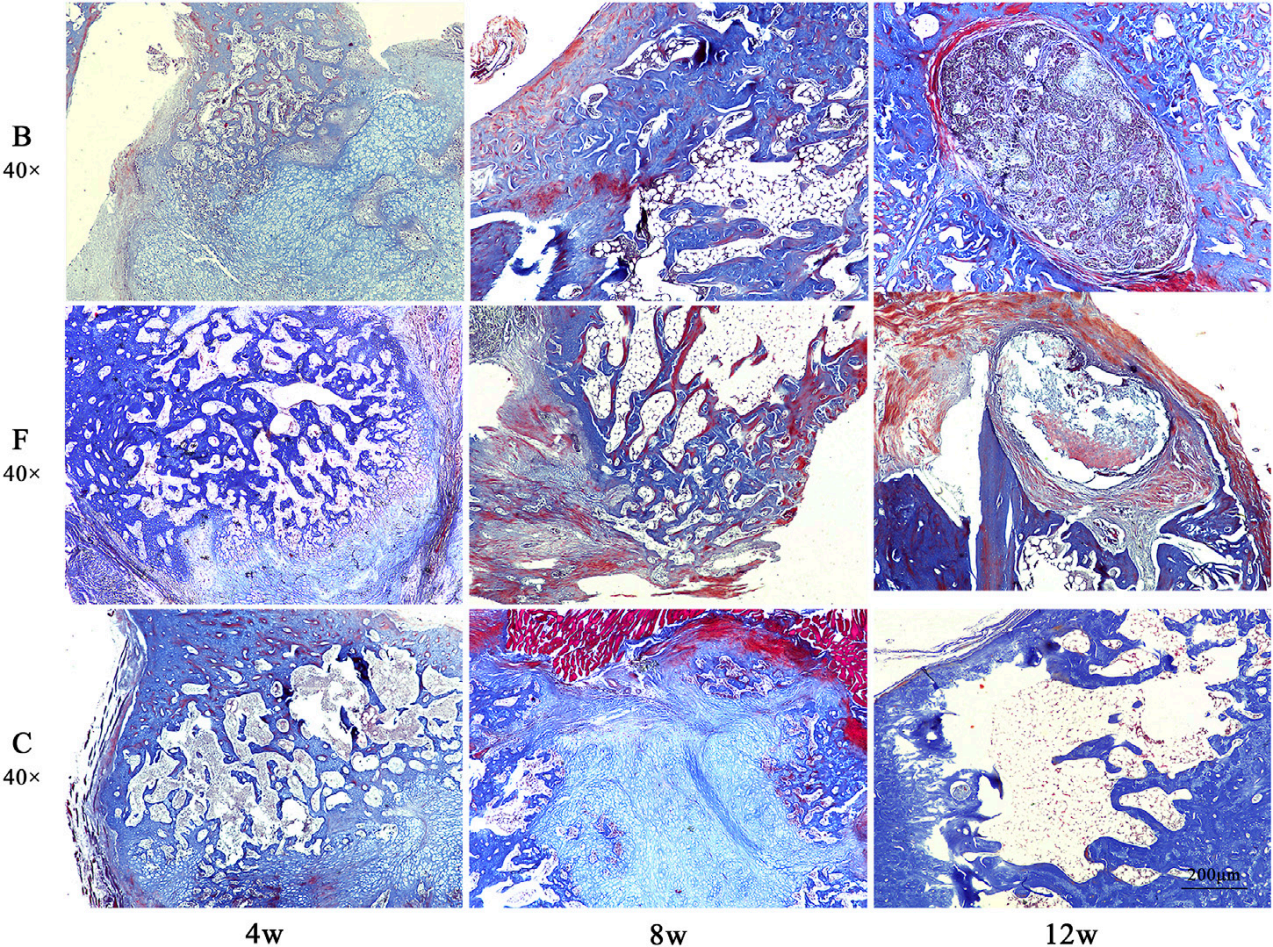
The Masson staining map of bone defects in rats. The tissues were taken for Masson staining at weeks 4, 8 and 12 after implantation (magnification, ×40).

Masson staining showed (Figure 14) that at week 4 postoperatively, all three groups of defect areas were heavily filled with collagen fibers internally. A portion of the FDBM and the BDBM were encapsulated by collagen fibers. In both bone material groups, there was some newly generated collagen and mature collagen from the edges of the defect area towards the interior, and the defect area was not clearly demarcated. In the self-healing group, there was no new collagen around or inside the defect area and the boundary with the original bone tissue was clearly visible. At week 8 postoperatively, there was a large amount of mature and new bone tissue filling the defect area in all three groups, but the filling density was significantly higher in the two bone material groups than in the self-healing group. At week 12 postoperatively, most of the bone defect area in the FDBM group had been replaced by new bone tissue, with only a small portion of the area remaining filled with relatively sparse bone tissue, which had not yet formed a mature and dense bone structure. In the bovine decalcified bone matrix group, a small area remained filled with collagen fibers, with a small amount of new bone tissue scattered within. In the self-healing group, most of the defect area was still filled with new bone tissue and collagen fibers, and no dense bone tissue was formed.

Combining the two staining results, it was found that the FDBM did not cause any immune response in the rats and had good biocompatibility. It also has good osteogenic induction, which can guide osteoblasts to grow in and form mature bone, new bone and osteoid at the edges of the defect area and within the material to fuse with the autologous femur. The FDBM group produced more new bone tissue and trabeculae in the early stages of repair than the BDBM group, and had better overall repair capacity. While new bone was formed, the material gradually degraded and had good degradation properties in the animals. The results show that the FDBM has the ability to be used as a bone repair material.

**FIGURE 15:**
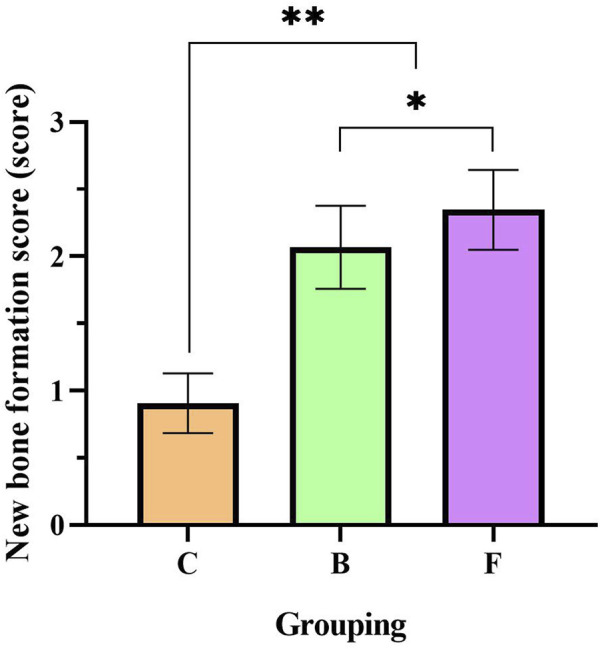
New bone formation score for femoral repair at 4 weeks. (C: Self-healing group; B: BDBM group; F: FDBM group) (**: Significant difference compared to the self-healing group, *p* < 0.01; *: Significant difference compared to BDBM, *p* < 0.05).

Semi-quantitative analysis showed (Figure 15) that the new bone formation score in the self-healing group was 0.9. Compared with the self-healing group, the new bone formation score was significantly higher in the DBM group (*p* < 0.01). And the FDBM group scoring slightly higher than the BDBM group (*p* < 0.05). With reference to the criteria of YY/T 1680–2020, it can be concluded that the FDBM has good osteoinductive potential.

## 4 Discussion

In reality, it is very common for people to suffer from bone defects as a result of work-related injuries, accidental injuries or injuries of medical origin. Currently, the implantation of bone tissue engineering repair materials remains the most effective method of treating patients with bone defects. Various types of bone tissue engineering materials have been widely used in clinical practice due to their excellent bone repair properties (Geng et al., 2022). However, there is still a problem of low osteogenic activity in clinical practice, which makes it difficult to meet the needs of a large number of clinical patients. In general, an ideal bone tissue engineering material should have sufficient osteoconductivity, osteoinductivity, good biocompatibility and degradability. DBM is a bone material that has been acid-treated to remove the mineral matrix, while retaining organic matter and growth factors. DBM was first reported to have an important osteogenic effect in 1965, with collagen as its main component and bone morphogenetic proteins (BMPs) with osteogenic activity but no species specificity as the rest. It is now generally accepted that the repair mechanism of implanted bone materials is to heal bone defects by promoting osteoblast growth and angiogenesis. DBM induces osteogenesis by removing the calcium salt barrier through decalcification, removing the encapsulation of calcium salts around BMPs and other osteogenic active factors and stimulating the conversion of MSCs into cartilage and osteoblasts. At the same time the decalcification process gives the DBM a natural pore structure. This not only allows the DBM to develop good plasticity, but also facilitates the slow release of BMPs and the growth of new bone and other tissues into it, thus improving the mechanical strength and enhancing the efficiency of the repair. And the antigenic surface structure of the DBM is disrupted during the acid treatment, resulting in no immune rejection and reduced morbidity at the surgical site. In our study, we use halibut fish bone that is left from the former procedure and obtained FDBM after a series of treatments, which effectively improved the utilization efficiency of fish bone and reduced solid waste.

### 4.1 Physicochemical properties and biocompatibility evaluation of FDBM

As a tissue engineering scaffold for bone defect repair medicine, it must have the characteristics of good porosity, mechanical strength, biocompatibility and biodegradability. The porosity of collagen materials increases with their internal surface area. During tissue repair, higher porosity of collagen materials can provide a wider space for cell adhesion, proliferation and differentiation. The results of scanning electron microscopy show that FDBM has a high porosity and basically meets the requirements of an ideal bone tissue engineering material. The mechanical strength is an important indicator to evaluate the mechanical properties of the scaffold, which indicates the effective load-bearing capacity of the mate rial and determines the tolerance to mechanical loading during the process from new tissue growth to the degradation of the scaffold matrix (Tao et al., 2017). The mechanical strength of the prepared FDBM is similar to that of human cancellous bone. It can withstand a large degree of deformation without rupture and basically meets the requirements of bone repair materials in terms of mechanical properties. The results showed that the treatment of incompletely decalcified can enable the scaffold to maintain a certain mechanical strength based on its good performance, which is beneficial for its application in bone tissue engineering. Additionally, because of its biodegradability, the FDBM can be de-graded by itself at the bone defects, so there is no need to take it out again, which reduces the occurrence of secondary trauma.

The rate of degradation of bone tissue engineered scaffolds is mainly determined by the nature of the material itself and the local physiological environment after implantation. *In vivo* degradation is mainly related to the action of osteoclasts and multinucleated macrophages. The rate of bone resorption by osteoblasts is higher than the rate of bone formation by osteoclasts, which can result in poor bone repair (Geng et al., 2021). During the formation of new bone, the scaffold material is broken down by components such as lysosomal enzymes released by osteoclasts. The residual fragments are engulfed by macrophages, thus allowing the scaffold material to be gradually degraded and resorbed. The results showed that the FDBM could be gradually degraded in the body, indicating it has good biosafety and biodegradability. Cell adhesion and spreading are two key factors in the regulation of cell functions (Geng et al., 2020). The results of the co-culture of cells and materials showed that the FDBM not only has high porosity, but also has good fibroblast adhesion and potential to induce fibroblast migration and growth, which fully indicates that the FDBM has good cell compatibility. Important means including hemolysis test, pyrogen test, subcutaneous implantation test, cytotoxicity test whether implanted bone tissue engineering materials are qualified. It was found that no adverse reactions occurred. The FDBM has an excellent biosafety profile and meets the requirements of a medical device.

### 4.2 Bone defect repair capacity of FDBM

The critical size defect (CSD) is the most commonly used model for evaluating materials for bone defect repair. Schmitz et al. define CSD as the smallest bone defect in a particular bone of a particular animal that does not heal over its lifetime (Gordon et al., 2008). Hollinger et al. define CSD as a bone defect that heals less than 10% over the life of the animal, and if this level is not reached within 1 year, the model is considered to meet the criteria for CSD (Gordon et al., 2008). However, most preclinical studies have a time limit for assessment and Gosia et al. state that “the critical size defect in animal studies is the size of the defect that does not heal during the study period” (Song et al., 2016). Female rats at 10 weeks of age were used for this experiment to ensure that the femur was of sufficient width. A 2 × 3 mm bone defect was created on the medial side of the upper middle femur of the rat. Throughout the experiment, the imaging and histological findings of the blank control group showed that the bone defect was not completely repaired and met the CSD criteria. The results show that the rat femoral bone defect model meets the requirements for discussing FDBM-based repair of femoral bone defects.

The repair of bone defects is a long and complex process. The safety and efficacy of the obtained FDBM were evaluated by bone defect repair testing in rats for 12 weeks, and compared with BDBM that have been used in clinical practice for many years. The results show that FDBM is slightly more effective than BDBM in repairing bone defects, and that it has a good *in vivo* biosafety profile, making it a promising medical biomaterial for the treatment of bone defects. It has been suggested that DBM alone has limited osteoinductive potential and does not have the ability to promote complete repair of bone defects (Giannoudis et al., 2005; Gerhardt et al., 2011; Yannas, 2013), and some experiments have demonstrated a lack of bone regeneration despite the use of DBM in critical size defects (Larranaga et al., 2014; Tainio et al., 2017). Up to 12 weeks postoperatively, the imaging results of the DBM group still showed incomplete healing of the bone defect, possibly due to partial loss and insufficient concentration of bone forming proteins during preparation, making it difficult to develop a dose effect. However, for the time being, the prepared FDBM is better than commercially available BDBM in the treatment of bone defects and has good biocompatibility. It also effectively improves the utilization of marine resources and reduces solid waste. In conclusion, FDBM is a promising medical biomaterial for the treatment of bone defects and is expected to replace BDMB in clinical practice.

## 5 Conclusion

In this study, we prepared FDBM from halibut fish bone and characterized its properties. The results showed that the FDBM has good porosity, mechanical strength, biodegradability and biocompatibility, which is conductive to cell infiltration, adhesion and growth. Its good bone repair ability was confirmed in a rat bone defect model, and it can effectively induce the growth of new bone tissue, and its repair speed and quality are better than those of the commercially available BDBM. The FDBM is an artificial bone material with good application prospects, which can basically meet the clinical requirements for bone tissue repair materials.

## Data Availability

The original contributions presented in the study are included in the article/supplementary material, further inquiries can be directed to the corresponding authors.
